# Deficiency of Cathelicidin Attenuates High-Fat Diet Plus Alcohol-Induced Liver Injury through FGF21/Adiponectin Regulation

**DOI:** 10.3390/cells10123333

**Published:** 2021-11-27

**Authors:** Fengyuan Li, Jenny Chen, Yunhuan Liu, Zelin Gu, Mengwei Jiang, Lihua Zhang, Shao-Yu Chen, Zhongbin Deng, Craig J. McClain, Wenke Feng

**Affiliations:** 1 Department of Medicine, University of Louisville, Louisville, KY 40202, USA; f0li0009@louisville.edu (F.L.); jjchen02@louisville.edu (J.C.); yunhuan.liu@louisville.edu (Y.L.); zelin.gu@louisville.edu (Z.G.); m0jian03@louisville.edu (M.J.); lhzhan01@louisville.edu (L.Z.); cjmccl01@louisville.edu (C.J.M.); 2 Department of Pharmacology and Toxicology, University of Louisville, Louisville, KY 40202, USA; s0chen23@louisville.edu; 3 Alcohol Research Center, University of Louisville, Louisville, KY 40202, USA; z0deng01@louisville.edu; 4 Hepatobiology & Toxicology Center, University of Louisville, Louisville, KY 40202, USA; 5 Robley Rex VA Medical Center, Louisville, KY 40202, USA

**Keywords:** cathelicidin, HFD plus alcohol, neutrophil, FGF21/adiponectin, adipose lipolysis

## Abstract

Alcohol consumption and obesity are known risk factors of steatohepatitis. Here, we report that the deficiency of CRAMP (cathelicidin-related antimicrobial peptide—gene name: *Camp*) is protective against a high-fat diet (HFD) plus acute alcohol (HFDE)-induced liver injury. HFDE markedly induced liver injury and steatosis in WT mice, which were attenuated in *Camp^–/–^* mice. Neutrophil infiltration was lessened in the liver of *Camp^–/–^* mice. HFDE feeding dramatically increased epididymal white adipose tissue (eWAT) mass and induced adipocyte hypertrophy in WT mice, whereas these effects were attenuated by the deletion of *Camp*. Furthermore, *Camp^–/–^* mice had significantly increased eWAT lipolysis, evidenced by up-regulated expression of lipolytic enzymes, adipose triglyceride lipase (ATGL) and hormone-sensitive lipase (HSL). The depletion of *Camp* also increased uncoupling protein 1 (UCP1)-dependent thermogenesis in the brown adipose tissue (BAT) of mice. HFDE fed *Camp^–/–^* mice had elevated protein levels of fibroblast growth factor 21 (FGF21) in the eWAT, with an increased adiponectin production, which had been shown to alleviate hepatic fat deposition and inflammation. Collectively, we have demonstrated that *Camp^–/–^* mice are protected against HFD plus alcohol-induced liver injury and steatosis through FGF21/adiponectin regulation. Targeting CRAMP could be an effective approach for prevention/treatment of high-fat diet plus alcohol consumption-induced steatohepatitis.

## 1. Introduction

It is well recognized that consumption of diet containing high level of fat or alcohol induces fatty liver disease through multiple mechanisms [1] including microbiota and gut-liver axis [2,3]. Moreover, consumption of high-fat diet (HFD) and alcohol binge drinking synergistically induces fat deposition and inflammation in the liver [4,5]. Higher serum ALT levels have often been found in obese alcoholics, who are usually at high risk of developing more advanced alcohol- associated liver disease (ALD) such as steatohepatitis, fibrosis, and cirrhosis [6,7,8,9,10]. Antimicrobial peptides are a unique and diverse group of molecules that help to maintain a balanced microbiota and are thus important in fatty liver disease. Cathelicidin-related antimicrobial peptide (CRAMP) is a unique antimicrobial peptide that functions also as an immunoregulator [11]. The role of CRAMP in HFD- or alcohol-induced fatty liver disease has been studied. Genetic deletion of CRAMP exacerbated, and overexpression or pharmacological CRAMP treatment inhibited, fatty liver and liver injury in HFD fed mice [12] or in chronic plus binge alcohol fed mice [13]. A recent study showed that feeding wild type mice with short- or long-term HFD plus a single binge ethanol synergistically upregulated chemokine CXCL1 expression, elevated free fatty acid levels in both serum and liver, increased neutrophil infiltration in the liver, eventually leading to exacerbated liver injury [14]. However, the role of CRAMP in HFD plus binge ethanol-induced liver injury is unknown.

Excess fat accumulation in the liver can occur as a result of increased fat uptake, enhanced fat synthesis through de novo lipogenesis (DNL), reduced fat oxidation and/or reduced fat export from the liver [15]. One of the major sources of fat contributing to hepatic steatosis is the non-esterified fatty acids (NEFA) that are released from white adipose tissue lipolysis [16,17]. Adipose tissue is an important endocrine organ that plays a critical role in energy homeostasis. Adiponectin is the most abundant adipokine produced by the adipose tissue and functions to regulate glucose and lipid metabolism in an endocrine manner [18,19,20]. Decreased circulating adiponectin levels have been associated with obesity in murine models and humans [21,22]. The regulation of adiponectin gene expression is tightly controlled by several transcription factors including peroxisome proliferator-activated receptor γ (PPARγ), the major positive regulator of adiponectin gene expression [23,24]. The activation of PPARγ also induces the expression of fibroblast growth factor 21 (FGF21), which has been shown to improve metabolic disorders in humans and mice [25,26]. Importantly, FGF21 enhances adiponectin secretion, which is essential for FGF21 to exert its metabolic effects [27]. Moreover, FGF21 prevents PPARγ sumoylation and restores its activity [28], partially contributing to adiponectin upregulation. Previously, we showed that the therapeutic effects of probiotic *Lactobacillus rhamnoses* GG (LGG) in mouse models of fatty liver diseases are mediated by FGF21/adiponectin upregulation [29,30]. However, the involvement of CRAMP in adipose tissue lipolysis and FGF21/adiponectin signaling has not been reported.

Here, we investigated the role of CRAMP in high-fat diet plus alcohol (HFDE)-induced liver injury and steatosis using WT and CRAMP KO mice. We explored the underlying mechanisms of CRAMP regulation in neutrophil infiltration, adipose lipolysis and FGF21/adiponectin signaling. We found that *Camp^–/–^* mice are protected against HFDE-induced liver fat accumulation and liver injury through suppressing hepatic neutrophil infiltration, increasing adipose lipolysis and enhancing FGF21/adiponectin production. 

## 2. Materials and Methods

### 2.1. Animals and Treatments

**Figure 1:**
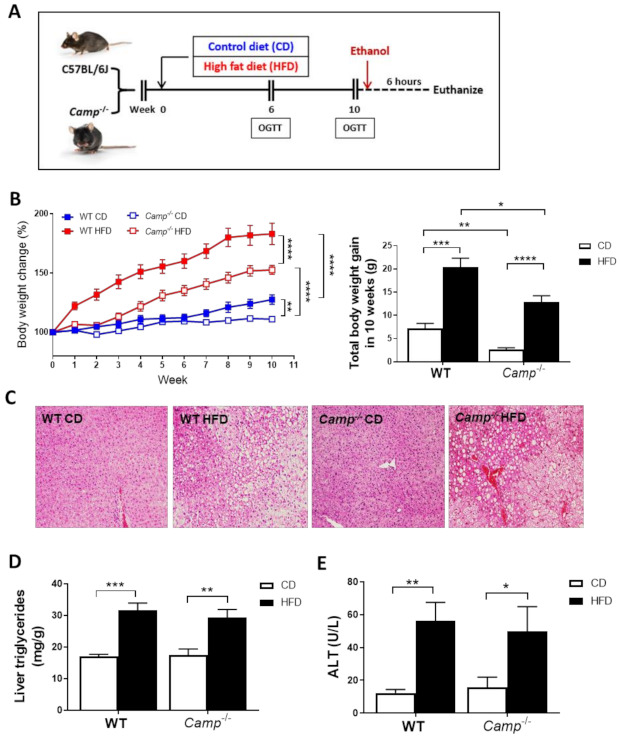
Feeding paradigm and the effects of *Camp* deletion on HFD-induced body weight gain, liver steatosis and injury. (**A**) Experimental design. (**B**) Body weight change of WT and *Camp^–/–^* mice with HFD feeding over 10 weeks (left panel). Total body weight gain in 10 weeks (right panel). (**C**) Representative images of H&E-stained liver tissues. Magnification: 100×. (**D**) Liver triglyceride contents. (**E**) Serum ALT levels. CD: control diet; HFD: high-fat diet. Data are expressed in Mean ± SEM (n = 4–9 mice/group). * *p* ≤ 0.05, ** *p* ≤ 0.01, *** *p* ≤ 0.001, **** *p* ≤ 0.0001.

CRAMP KO (*Camp*^−/−^) male mice (Jackson Laboratory, Bar Harbor, ME) at the age of 8–10 weeks were used with their age-matched WT mice. Mice were maintained at 22 °C with a 12 h light/dark cycle and had free access to a normal chow diet and tap water. WT and Camp^–/–^ mice were subjected to an adjusted calories control diet (CD) (10% total kcal from fat) or an adjusted calories high-fat diet (HFD) (42% total kcal from fat) for 10 weeks, followed by one binge alcohol gavage (CDE/HFDE), 6 h before tissue harvesting. The feeding paradigm is depicted in Figure 1A. During the 10-week feeding period, mouse body weights in each feeding group were recorded weekly. At the end of the 6th and 10th week, an oral glucose tolerance test (OGTT) was conducted in each CD or HFD feeding group. At harvest, a portion of the liver and eWAT tissue were collected and fixed in formalin solution for histological analysis, and the rest of the tissues were stored for mRNA and protein extraction. The serum samples were collected for biochemical study. 

### 2.2. ALT and AST Measurements

Serum activity levels of alanine aminotransferase (ALT) and aspartate aminotransferase (AST) were determined kinetically using standard kits (Thermo Scientific, Waltham, MA, USA).

### 2.3. Histological Analysis

#### 2.3.1. H&E Staining

Liver tissue and epidydimal white adipose tissue (eWAT) were collected and fixed in 4% paraformaldehyde and embedded in paraffin. The paraffin-embedded liver tissue blocks were then sliced at 5 µm thickness and eWAT tissue sliced at 7 µm on a microtome. The sliced tissues were then floated in a water bath containing distilled water at 45 °C for the liver tissue and at 40 °C for the adipose tissue. Sections were then transferred onto glass slides. Slides were air dried for 2 days or dried in a 37 °C incubator overnight before the day of staining, otherwise were stored at room temperature for future use. Slides were deparaffinized with Citrisolv (Decon, King of Prussia, PA, USA) and rehydrated by immersing in graded ethanol solutions before staining. Hematoxylin and eosin (H&E) were used to stain the nuclear and cytoplasm components, respectively. After staining, sections were then dehydrated through graded alcohol, cleared in Citrisolv and then mounted with Cytoseal Xyl (Thermo Scientific, Waltham, MA, USA) and observed by microscopy.

#### 2.3.2. CAE Staining

Infiltration of neutrophils in the liver was assessed by chloroacetate esterase (CAE) staining according to manufacturer’s instructions (Naphthol AS-d Chloroacetate Kit, Sigma, St. Louis, MO, USA). Briefly, liver sections were incubated in a solution of naphthol AS-D chloroacetate, the naphthol AS-D chloroacetate is enzymatically hydrolyzed by “specific esterase” in neutrophils, liberating a free naphthol compound. This, then, couples with a diazonium compound, forming highly colored deposits at sites of enzyme activity that can be visualized under microscopy.

#### 2.3.3. Ly6G and CRAMP Colocalization Immunohistochemistry

Following deparaffinization, antigen retrieval was performed on slides in 10 mM citrate buffer (pH6.0) for 20 min at 95 °C. Blocking buffer containing 10% fetal bovine serum (FBS) in PBS (Sigma, St. Louis, MO, USA) was added onto the slides and the slides were incubated in a humidified chamber at room temperature for 1 h, for the blocking of non-specific antibody binding. Sections were then incubated with primary antibody Ly6G (rat-anti-mouse), cathelicidin (mouse CRAMP, rabbit-anti-mouse) (Abcam, Cambridge, UK) at a concentration of 1:200 in 10% FBS and incubated in a humidified chamber overnight at 4 °C. On the next day, slides were washed with PBS for 3 times, 1 min/time before being incubated with AlexaFluor-conjugated secondary antibodies (Life technologies, Carlsbad, CA, USA). AlexaFluor 488 (Green) (donkey-anti-rat) and AlexaFluor 594 (Red) (goat-anti-rabbit) at a concentration of 1:200 in 10% FBS were used to incubate the slides for 1.5 h. DAPI (Invitrogen, Carlsbad, CA, USA) was used for nuclei counterstain. The fluorescence was examined under confocal microscopy and the intensity of fluorescence was quantified using Image J.

### 2.4. Oral Glucose Tolerance Test (OGTT)

At week 6 and week 10, OGTTs were performed for both CD and HFD fed WT and CRAMP KO mice. Methods used are as described [31]. Briefly, mice were fasted overnight for about 16 h and weighed to calculate the glucose dose at 2.5 g/kg body weight. The working glucose solution was prepared at concentration of 250 mg/mL in advance. Blood glucose level of each mouse was determined by glucometer in tail vein blood before the glucose gavage (T0) and at 15 (T15), 30 (T30), 60 (T60) and 120 (T120) minutes after that. Tail snipping is used to get blood. Before snips, the tail end was dipped into Bupivacaine (0.25%) for local anesthesia to reduce pain.

### 2.5. Lipid Accumulation

Liver tissues were homogenized in 50 mM NaCl (Sigma, St. Louis, MO, USA) solution in distilled water. The homogenates were then used for lipid extraction using methanol (Fisher chemical, Fair Lawn, NJ, USA) and chloroform (Fisher chemical, Fair Lawn, NJ). Liver triglyceride (TG), free fatty acid (FFA) levels and total cholesterol levels were determined using commercial kits according to the manufacturer’s instructions (Thermo Scientific, Waltham, MA, USA).

### 2.6. RNA Isolation and Real-Time RT-PCR

The mRNA levels were assessed by real-time polymerase chain reaction (PCR). In brief, the total RNA was extracted from liver, colon, spleen, epididymal white adipose tissue (eWAT) and lung tissue with Trizol according to the manufacturer’s protocol (Life technologies, Carlsbad, CA, USA) and reverse-transcribed using cDNA supermix (QuantaBio, Beverly, MA, USA). Quantitative real-time PCR was performed on an ABI 7500 real-time PCR thermocycler, whereas SYBR green PCR Master Mix (Applied Biosystems, Foster City, CA, USA) was used for real-time PCR analysis. The relative quantities of target transcripts were calculated from duplicate samples after normalization of the data against the housekeeping gene, 18S rRNA (mouse). Relative mRNA expression was calculated using the delta-delta Ct method. The primer pairs used were listed in Table S1. 

### 2.7. Immunoblots

Liver and eWAT tissues were lysed in radioimmunoprecipitation assay (RIPA) buffer (Thermo Scientific, Waltham, MA, USA) containing 50 mM Tris·HCl, 150 mM NaCl, 2 mM EDTA, 4 mM Na3VO4, 40 mM NaF, 1% Triton X-100, 1 mM PMSF, 1% protease inhibitor cocktail. Lysed samples were then centrifuged for 15 min at 12,000× *g* at 4 °C, and the supernatants were collected. The protein concentration was measured by using a bicinchoninic acid assay (BCA) assay kit (Pierce, Thermo Scientific, Waltham, MA, USA). Sample aliquots were boiled for 5 min and equal protein amounts (usually 35–50 μg) were separated by SDS-PAGE. Proteins were then transferred to a nitrocellulose membrane. Blots were blocked for 1 h in Tris-buffered saline/Tween 20 (TBST buffer) (10 mM Tris–HCl, pH 8.0, 150 mM NaCl, and 0.05% Tween 20) containing 5% nonfat dry milk and incubated overnight at 4 °C with the different primary antibodies diluted (typically 1:1000; ACTβ at 1:10,000) in TBST buffer containing 3% BSA. Blots were then incubated in horseradish peroxidase (HRP)-conjugated secondary antibodies (typically 1:5000 dilution) for 1 h at room temperature. Following treatment with supersignal west HRP substrate (MilliporeSigma, Billerica, MA, USA), protein bands were detected by a ChemiDoc Molecular Imager (Bio-Rad, Hercules, CA, USA). Quantification for the bands was performed using ImageJ software (National Institutes of Health) and normalized to ACTβ. The following primary antibodies were used: FASN (Cell signaling, Danvers, MA, USA), CRAMP (Abcam, Cambridge, UK), ATGL (Cell signaling, Danvers, MA, USA), pHSL (Cell signaling, Danvers, MA, USA), HSL (Cell signaling, Danvers, MA, USA), UCP1 (Cell signaling, Danvers, MA, USA), FGF21 (Abcam, Cambridge, UK), PPARγ (Cell signaling, Danvers, MA, USA), ACTβ (Cell signaling, Danvers, MA, USA).

### 2.8. Serum TNFα, IL6, FGF21, Adiponectin ELISA

Mouse serum protein levels of TNFα and IL6 were determined using ELISA kits (Invitrogen, Carlsbad, CA, USA) according to the manufacturer’s instructions. Serum FGF21 protein levels were determined using Mouse/Rat FGF21 Quantikine ELISA Kit (R&D, Minneapolis, MN, USA) according to the manufacturer’s instructions. Serum adiponectin levels were determined using Mouse Adiponectin ELISA Kit (Abcam, Cambridge, UK).

### 2.9. Endotoxin Assay

Chromogenic limulus amebocyte lysate (LAL) endotoxin kit was used to determine serum endotoxin levels according to the manufacturer’s protocol (Lonza, Basel, Switzerland). All material used for harvesting blood and measuring endotoxin was pyrogen free.

### 2.10. Flow Cytometry

Hepatic leukocytes were prepared as previously described [32]. Briefly, a portion of the liver tissue was collected immediately upon harvest and was homogenized with a 70-μm pore filter in complete DMEM with 2% FBS on ice. The homogenates were then centrifuged at 2300 rpm for 15 min. After washing extensively, the homogenates were resuspended in a 33% Percoll gradient at 22 °C and centrifuged at 2300 rpm for 20 min. The supernatant with the upper layer, which contains the dead hepatocytes, was discarded and the cell pellet was collected. Red blood cells (RBCs) were then removed using red cell lysis buffer followed by a wash with 1xPBS and centrifugation. Isolated hepatic leukocytes were blocked by incubating with CD32 antibody, then labeled with antibodies using standard procedures as described previously [33]. Gr1, CD11b and Ly6C antibodies that are conjugated with PerCP, APC and FITC from eBioscience (San Diego, CA, USA) were used. Cells were analyzed by flow cytometry using a FACScan (BD Biosciences protocol; BD Pharmingen). Histogram analysis was performed using FlowJo Software (Tree Star, Ashland, OR, USA).

### 2.11. Statistical Analysis

Statistical analyses were performed using the statistical computer package, GraphPad Prism version 6 (GraphPad Software Inc., San Diego, CA, USA), MS Excel 2016 (Microsoft Corp., Redmond, WA, USA) or SPSS 26.0 (IBM, Chicago, IL, USA). Results are expressed as mean ± standard error of the mean (SEM). Statistical comparisons were made using two-way analysis of variance (ANOVA) with Tukey’s post hoc test or Student’s *t*-test where appropriate. Differences were considered to be significant at *p* ≤ 0.05. Significance was noted as * *p* ≤ 0.05, ** *p* ≤ 0.01, *** *p* ≤ 0.001, **** *p* ≤ 0.0001 between groups. Results are presented as Mean ± SEM.

## 3. Results

### 3.1. Deletion of CRAMP Attenuated HFD-Induced Body Weight Gain but Had No Effects on HFD-Induced Liver Steatosis and Injury

Ten-weeks HFD feeding (Figure 1A) markedly increased the body weight of both WT and *Camp^–/–^* mice over time compared to those paired animals fed with a control diet (CD) (Figure 1B). However, the increase in body weight was more robust in WT mice (183.2% by HFD vs. 127.7% by CD) compared to *Camp^–/–^* mice (152.7% by HFD vs. 111.7% by CD). Importantly, *Camp^–/–^* mice grew at a slower rate overall compared to WT mice, in both CD and HFD feeding groups (Figure 1B). To determine whether CRAMP deficiency affects glucose homeostasis, we performed oral glucose tolerance tests (OGTT) at week 6 and week 10. Fasting glucose concentrations were not significantly different between CD and HFD-fed WT mice at week 6. However, HFD feeding significantly increased fasting serum glucose level in *Camp^–/–^* mice, compared to their CD controls and to HFD WT controls (Figure S1A). HFD feeding did not increase glucose intolerance at week 6 in either WT or *Camp^–/–^* mice. Surprisingly, *Camp^–/–^* mice showed increased glucose intolerance compared to their WT controls, and this was independent of the CD or HFD feeding regimen (Figure S1B). The fasting blood glucose level in WT and *Camp^–/–^* mice fed with HFD significantly increased by week 10 (Figure S1C), while 10-week HFD-fed *Camp^–/–^* mice had comparable fasting glucose levels to week 6 (Figure S1A,C). OGTT revealed that glucose tolerance was substantially decreased by HFD feeding in WT but not *Camp^–/–^* mice at week 10 (Figure S1D). These results indicated that HFD feeding increased fasting glucose concentration in *Camp^–/–^* mice at an early stage but attenuated it at a later stage. In contrast, HFD increased fasting glucose level and induced glucose intolerance in WT mice over time. HFD feeding induced liver steatosis in both WT and *Camp^–/–^* mice to a comparable level, evidenced by histological analysis of the liver tissue (Figure 1C), which was further confirmed by the measurement of hepatic triglyceride content (Figure 1D). There were no changes on HFD-induced liver injury between WT and *Camp^–/–^* mice, shown as serum ALT levels (Figure 1E). We thus focused on the groups with ethanol gavage (CDE/HFDE) for the following studies. 

### 3.2. Deletion of CRAMP Decreased HFD Feeding Plus Ethanol-Induced Liver Injury and Steatosis

**Figure 2:**
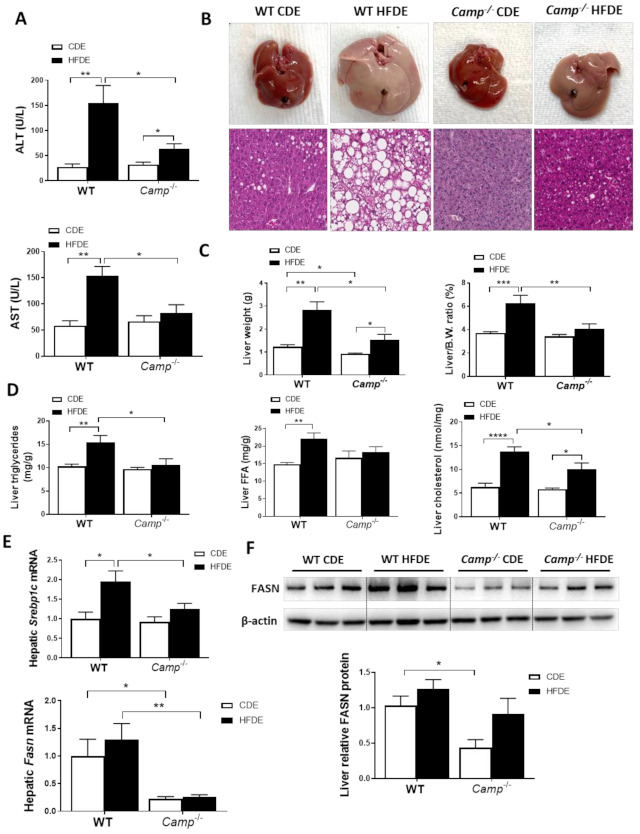
*Camp^–/–^* mice had significantly less injury and fat accumulation in the liver. (**A**) Serum ALT and AST levels. (**B**) Images of representative whole liver (upper panel) and microphotographs of H and E-stained mouse liver sections (lower panel). Magnification: 100×. (**C**) Liver weight (left panel) and liver to body wight ratio (right panel). (**D**) Hepatic triglyceride, free fatty acid and total cholesterol levels. (**E**) Hepatic *Srebp1c* and *Fasn* mRNA levels. (**F**) Representative immunoblots of FASN protein in the liver tissue and quantification of relative protein expression to ACTβ. Data are expressed in Mean ± SEM (n = 5 mice/group). * *p* ≤ 0.05, ** *p* ≤ 0.01, *** *p* ≤ 0.001, **** *p* ≤ 0.0001.

HFDE treatment markedly induced liver injury in WT mice, as reflected by significantly increased serum levels of ALT and AST, while only a moderate increase in serum AST was observed in *Camp^–/–^* mice (Figure 2A). HFDE fed WT mice had dramatically enlarged livers compared to their CDE controls and HFDE fed *Camp^–/–^* mice (Figure 2B). Histological analysis revealed dramatically increased liver macrosteatosis in HFDE fed WT mice, while only mild microsteatosis was found in the livers of *Camp^–/–^* mice (Figure 2B). The liver weight was significantly increased by HFDE treatment in both WT and *Camp^–/–^* mice, but to a much lesser extent in *Camp^–/–^* mice (Figure 2C). The liver/body weight ratio markedly increased by HFDE in WT mice compared to CDE, whereas no significant differences between HFDE and CDE were found in *Camp^–/–^* mice (Figure 2C). Confirming these results, we showed significantly increased liver triglyceride concentration, free fatty acid content, and total cholesterol level in WT mice by HFDE, which were attenuated in *Camp^–/–^* mice (Figure 2D). Hepatic de novo lipogenesis (DNL) is a fundamental biosynthetic pathway that contributes to hepatic lipid accumulation. We found that HFDE significantly increased mRNA expression of *Srebp1c*, a key transcription factor in DNL [34] in the livers of WT mice, but not in *Camp^–/–^* mice (Figure 2E). We further analyzed the expression of fatty acid synthase (FASN), the rate-limiting enzyme in DNL and a transcriptional target of SREBP1c [35]. HFDE slightly increased FASN expression on both mRNA (Figure 2E) and protein levels (Figure 2F) in WT mice. However, the deletion of CRAMP significantly decreased hepatic *Fasn* mRNA expression regardless of CDE or HFDE feeding, and moderately reduced FASN protein level in the livers of HFDE treated mice (Figure 2E,F). Collectively, CRAMP deficiency decreased HFD plus acute alcohol-induced liver injury and hepatic lipid accumulation.

### 3.3. Camp Depletion Normalized the Increase in the Inflammatory Response Induced by HFDE

**Figure 3:**
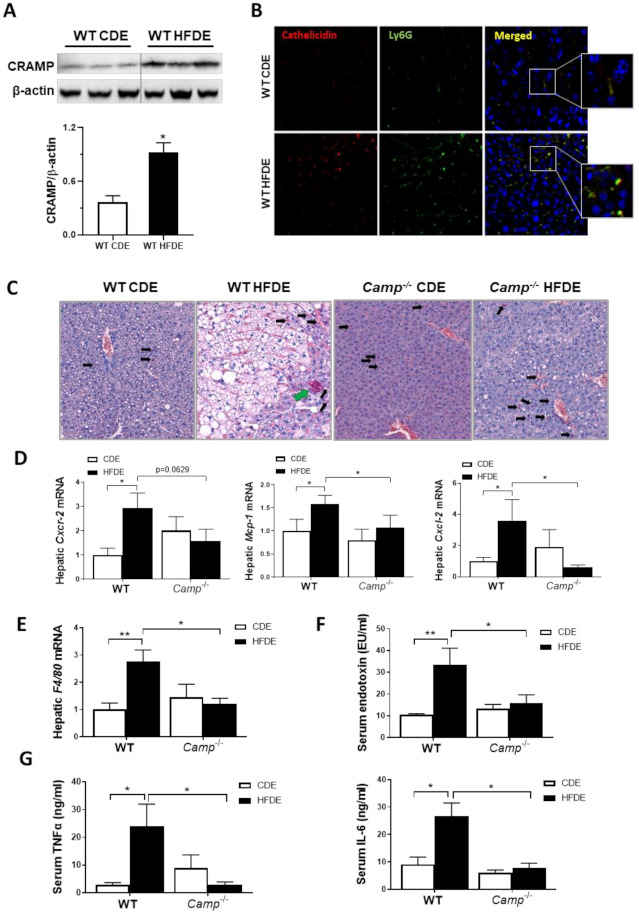
*Camp^–/–^ mice* had attenuated inflammatory response induced by HFDE treatment in the liver. (**A**) Representative immunoblots (upper panel) and quantification (lower panel) of CRAMP protein in whole liver lysates of WT mice. (**B**) Immunofluorescence (IF) co-staining of Ly6G (Green) and cathelicidin (antibody for mouse CRAMP) (Red) antibodies. Blue: DAPI for nucleus staining. Magnification: 200×. (**C**) CAE staining of paraffin embedded liver tissue. Infiltrated scattered neutrophils: black arrows; clustered neutrophils: green arrow. Magnification: 100X. (**D**) Hepatic mRNA level of *Cxcr2*, *Mcp1* and *Cxcl2*. (**E**) Hepatic *F4/80* mRNA level. (**F**) Serum endotoxin concentration. (**G**) Serum levels of TNFα and IL6. Data are expressed in Mean ± SEM (n = 5 mice/group). * *p* ≤ 0.05, ** *p* ≤ 0.01.

We have previously shown that acute alcohol exposure increased hepatic CRAMP expression on both mRNA and protein levels in WT mice primed with 24 days of chronic ethanol feeding [13]. We thus evaluated CRAMP expression induced by acute ethanol in 10-week HFD-primed livers of WT mice. Hepatic *Camp* mRNA was not affected by HFDE (Figure S2A), but the protein level of CRAMP significantly increased in the livers of WT mice (Figure 3A). Flow cytometry analysis of hepatic leukocytes revealed a significantly increased population of inflammatory monocytes in the livers of WT mice fed with HFDE (Figure S2B). Immunofluorescence staining of liver tissue from WT mice fed CDE or HFDE revealed a colocalization of CRAMP and Ly6G-positive neutrophils, which were both increased by HFDE feeding (Figure 3B). These results suggested an enhanced neutrophil infiltration and chemokine expression by acute ethanol in HFD-primed liver, which is possibly CRAMP-dependent. CAE staining showed a robust reduction in neutrophil infiltration in the livers of *Camp^–/–^* mice fed with HFDE compared to WT (Figure 3C) mice. Of note, the infiltrated neutrophils in the liver of HFDE fed *Camp^–/–^* mice were scattered, whereas neutrophils formed as clusters in the livers of WT mice (differentiated by black arrows and green arrow in Figure 3C, respectively). Human cathelicidin LL37 has been reported to act as a functional ligand for chemokine receptor CXCR2 on human neutrophils [36]. We thus analyzed the mRNA level of murine *Cxcr2*. As expected, acute ethanol significantly up-regulated *Cxcr2* in HFD-primed livers in WT but not *Camp^–/–^* mice (Figure 3D). Importantly, feeding *Camp^–/–^* mice with HFDE moderately reduced *Cxcr2* mRNA expression. In addition, hepatic mRNA expression of chemokines *Mcp1* and *Cxcl2* was significantly increased in the livers of HFDE-fed WT mice, and was reduced by the depletion of *Camp* gene (Figure 3D). Furthermore, we found that *Camp^–/–^* mice had decreased mRNA expression of the macrophage marker, *F4/80*, in the liver compared to HFDE fed WT mice (Figure 3E). Lipopolysaccharides (LPS) or endotoxin, released from the outer membrane of Gram-negative bacteria, has been shown to interact with macrophages to induce the release of endogenous pro-inflammatory mediators, such as TNFα and IL6 [37]. We found that serum endotoxin level was significantly increased by HFDE treatment in WT mice, but was normalized by *Camp* depletion (Figure 3F). As expected, the serum protein levels of TNFα and IL6 were robustly decreased in HFDE fed *Camp^–/–^* mice compared to their WT controls (Figure 3G).

### 3.4. The Deletion of CRAMP Significantly Inhibited Adipocyte Hypertrophy and Attenuated Adipose Tissue Inflammation Induced by HFDE Treatment

**Figure 4:**
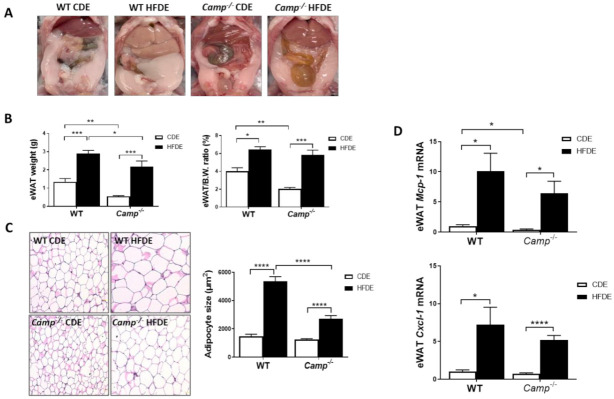
*Camp* deletion significantly inhibited adipocyte hypertrophy and moderately attenuated adipose tissue inflammation induced by HFDE. (**A**) Epididymal adipose tissue shown in abdominal cavity image of mice. (**B**) eWAT weight (left panel) and eWAT/body weight ratio (right panel). (**C**) Representative microphotographs of H&E-stained mouse eWAT sections (left panel) and the quantification of adipocyte size (right panel). Magnification: 200×. (**D**) mRNA levels of eWAT *Mcp1* and *Cxcl1*. Data are expressed in Mean ± SEM (n = 5 mice/group). * *p* ≤ 0.05, ** *p* ≤ 0.01, *** *p* ≤ 0.001, **** *p* ≤ 0.0001.

HFD feeding induces obesity and metabolic syndrome, which are usually characterized by an expansion of white adipose tissue (WAT) and adipocyte hypertrophy [38,39,40]. We observed a dramatic increase in epididymal white adipose tissue (eWAT) mass in HFDE fed WT mice, compared to their CDE fed controls and HFDE fed *Camp^–/–^* mice (Figure 4A). We thus evaluated the differences in body fat mass by determining the ratio of epididymal adipose tissue weight (eWAT) to body weight. *Camp^–/–^* mice had reduced eWAT weight and eWAT/body weight ratio under CDE, compared to the WT mice. HFDE significantly increased the weight of eWAT and the ratio of eWAT/body in both WT and *Camp^–/–^* mice (Figure 4B). Histological analysis of the adipose tissue showed a markedly increased adipocyte size in both WT and *Camp^–/–^* mice by HFDE feeding, but to a much-lessened extent in the *Camp^–/–^* mice (Figure 4C). Expansion of WAT and hypertrophy of adipocytes have been associated with adipose inflammation [41], we thus examined whether CRAMP is associated with HFDE-induced eWAT inflammation. HFDE robustly increased the mRNA expression of *Mcp-1* and *Cxcl-1* in WT mice, which were moderately lowered in the *Camp^–/–^* mice (Figure 4D). 

### 3.5. The Deletion of CRAMP Increased eWAT Lipolysis and Lipid Utilization by Brown Adipose Tissue, and Decreased Hepatic Fatty Acid Uptake in Mice Fed with HFD Plus Ethanol

**Figure 5:**
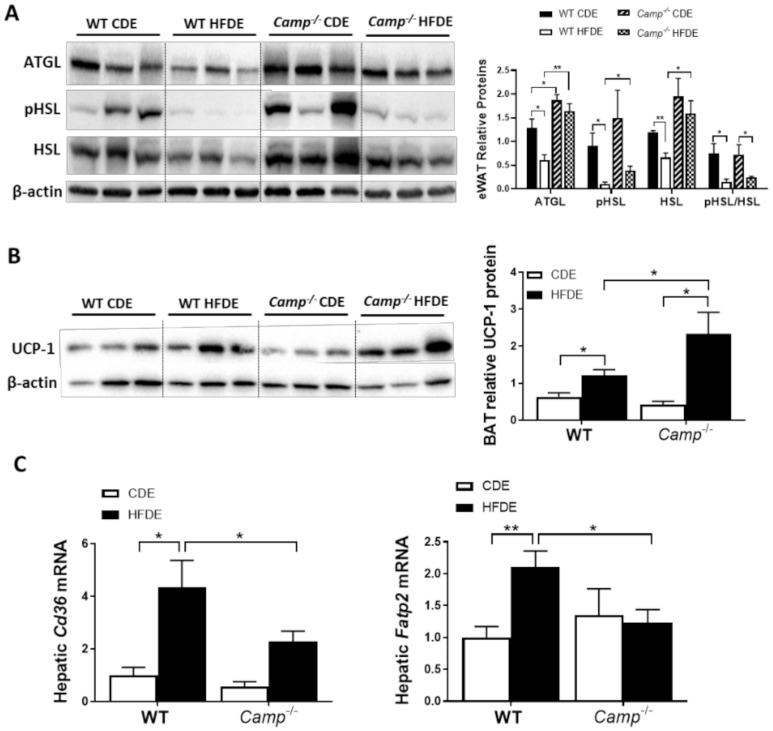
The effects of *Camp* deletion on adipose lipolysis, UCP1 protein expression in brown adipose tissue, and hepatic lipid transporter gene expression in mice fed with HFDE. (**A**) Representative immunoblots (left panel) and quantification (right panel) of lipolytic enzymes ATGL and HSL in eWAT. (**B**) Representative immunoblots (left panel) and quantification (right panel) of BAT UCP1 protein. (**C**) Hepatic mRNA expression of *Cd36* and *Fatp2*. Data are expressed in Mean ± SEM (n = 5 mice/group). * *p* ≤ 0.05, ** *p* ≤ 0.01.

Impaired lipolysis has been shown to contribute to the development of HFD-induced obesity via adipocyte hypertrophy in white adipose tissue, which is associated with a decreased expression of adipose triglyceride lipase (ATGL) [42], a key enzyme responsible for the lipolytic process. We thus examined the expression of lipolysis markers, including ATGL in eWAT tissue by immunoblotting. HFDE significantly decreased eWAT ATGL protein expression in WT but not *Camp^–/–^* mice, which had higher ATGL levels under CDE feeding compared to the WT mice (Figure 5A). Hormone-sensitive lipase (HSL), another critical lipolytic enzyme, was significantly reduced in the eWAT of WT mice by HFDE, but not in the *Camp^–/–^* mice. Phosphorylation of HSL was markedly reduced by HFDE in WT mice, and this reduction was also observed in KO mice but to a much lesser extent (Figure 5A). These results suggested increased adipose tissue lipolysis when CRAMP is depleted, which could contribute to the attenuated adipocyte hypertrophy observed in HFDE fed *Camp^–/–^* mice, while at the same time possibly leading to increased circulating free fatty acids (FFA). However, serum FFA levels were not affected by the deletion of *Camp* under HFDE feeding (Figure S3). Interestingly, we found that *Camp^–/–^* mice had increased level of uncoupling protein 1 (UCP1) in the brown adipose tissue (BAT) (Figure 5B), indicating that the utilization of lipids is increased when *Camp* is deleted. Moreover, *Camp^–/–^* mice had a decreased hepatic mRNA expression of fatty acid transporters, CD36 and fatty acid transport protein 2 (FATP2) (Figure 5C), suggesting that the uptake of fatty acids by the liver is inhibited when CRAMP is knocked out, which is in line with the decreased hepatic fat accumulation in *Camp^–/–^* mice. Taken together, these results indicate that CRAMP deficiency increases adipose lipolysis and lipids usage by BAT, while inhibiting hepatic lipids uptake, which together contribute to the attenuated hepatic steatosis.

### 3.6. HFD Feeding Plus Ethanol Promoted FGF21/Adiponectin Upregulation in Camp^–/–^ Mice

**Figure 6:**
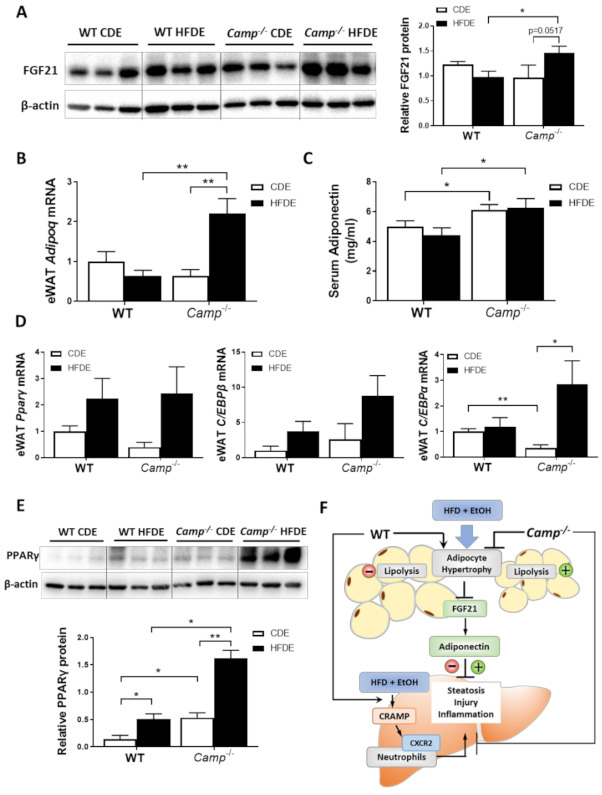
HFD feeding plus alcohol promoted FGF21/adiponectin upregulation in *Camp^–/–^* mice. (**A**) Representative images of eWAT FGF21 immunoblotting (left panel) and its quantification (right panel). (**B**) eWAT *Adipoq* mRNA levels. (**C**) Serum adiponectin protein levels, n = 6–9. (**D**) Relative mRNA level of *Pparg*, *Cebpb* and *Cebpa* in eWAT. (**E**) Representative Western blots for PPARγ protein level (upper panel) and quantification (lower panel) in eWAT. (**F**) Schematic hypothesis of the protective effects of CRAMP deficiency in HFDE-induced liver steatosis, injury and inflammation. Data are expressed in Mean ± SEM (n = 5 mice/group). * *p* ≤ 0.05, ** *p* ≤ 0.01.

Previous studies by our group have demonstrated that alcohol-induced adipose lipolysis is mediated by FGF21 [17]. FGF21 functions in the adipose tissue to induce adiponectin expression, which, in turn, reduces hepatic fat accumulation [29]. To explore whether the inhibitory effects of CRAMP on adipose lipolysis are mediated by the FGF21-adiponectin signaling pathway, we examined the expression of FGF21 in the livers and eWAT tissue of WT and *Camp^–/–^* mice. Immunoblots showed that HFDE increased the FGF21 protein levels in the eWAT of *Camp^–/–^* mice, but this effect was not observed in WT mice (Figure 6A). No significant changes were found on the mRNA level of eWAT FGF21 in both WT and *Camp^–/–^* mice (Figure S4). We further examined the expression of FGF21 in the liver, which is the major organ for circulating FGF21 production. HFDE increased the liver FGF21 protein in both WT and *Camp^–/–^* mice (Figure S5A). Interestingly, we found that serum FGF21 protein was moderately reduced by HFDE in WT and *Camp^–/–^* mice (Figure S5B). It has been shown that HFD or alcohol alone induces circulating FGF21 elevation [17,43]. However, the combination of HFD and alcohol may inhibit the release of FGF21 that is produced in the liver, and this inhibition was partially recovered by the deletion of *Camp*. Adiponectin is an adipokine that regulates lipid metabolism and inflammation in multiple organs, including the liver. It is well-known that adipose-derived FGF21 acts locally to stabilize adiponectin expression [29,30]. We found that the mRNA expression of adipose tissue adiponectin encoding gene *Adipoq* was moderately decreased in the WT mice, but was significantly increased in *Camp^–/–^* mice by HFDE (Figure 6B). Similarly, serum adiponectin levels were slightly decreased in the WT mice. Interestingly, *Camp^–/–^* mice had markedly elevated serum adiponectin levels under both CDE and HFDE conditions compared to WT mice (Figure 6C). The mRNA levels of transcription factors that regulate *Adipoq* expression were examined. The mRNA levels of PPARγ and CCAAT-enhancer-binding protein β (CEB/Pβ) showed a trend of increase by HFDE treatment in both WT and *Camp^–/–^* mice (Figure 6D). Interestingly, *CEB/Pα* mRNA levels were significantly decreased by CDE, but significantly increased by HFDE treatment in *Camp^–/–^* mice (Figure 6D). We further analyzed the protein level of PPARγ in eWAT by immunoblotting. HFDE increased eWAT PPARγ protein level in both WT and *Camp^–/–^* mice, but more robustly in *Camp^–/–^* mice. Moreover, *Camp^–/–^* had significantly higher levels of PPARγ protein compared to WT mice under both CDE and HFDE conditions (Figure 6E). These data suggest that the deletion of *Camp* upregulated FGF21/adiponectin signaling in mice under HFD plus alcohol treatment. 

## 4. Discussion

Excessive alcohol intake and obesity are common across the globe and have long been a public health concern. A combination of binge alcohol intake and a high fat-containing diet has been shown to synergistically promote the development and progression of alcohol-related liver diseases in patients [7,44] and animal models [45,46,47,48,49,50]. However, the underlying mechanisms have not been extensively studied. Recently, studies of antimicrobial peptides (AMPs) in metabolic diseases have emerged, as these small peptides also possess immunomodulating activity [51,52,53,54,55] in addition to their microbicidal potential [56,57,58]. Previous studies have demonstrated the critical role of AMPs in the pathogenesis of ALD through inhibiting bacteria overgrowth and endotoxemia [59,60]. A more recent study by Chang et al. showed that mice primed with an HFD feeding had exacerbated liver injury upon binge alcohol administration, and that the synergistic effects of HFD and alcohol are mediated by increased chemokine expression and neutrophil infiltration [14]. 

The only member of the human cathelicidin AMP family, LL37, has been shown to function as a chemoattractant to promote chemokine production and neutrophil infiltration [61]. On the other hand, LL37 also exhibits anti-inflammatory potential, as it has been shown to inhibit LPS binding to macrophages [62]. We have previously shown in a mouse model of ALD that lacking CRAMP, the murine ortholog of LL37, exacerbated binge-on chronic alcohol-induced liver injury and steatosis through inhibiting LPS and uric acid-mediated inflammasome activation [13], however, the role of CRAMP/LL37 in alcohol-induced liver injury in high-fat-diet-primed animals has not been described. 

In the present study, we demonstrated a protective effect of *Camp* deletion against HFDE-induced liver injury and steatosis in mice. Specifically, we showed that mice lacking CRAMP (*Camp^–/–^*) had greatly attenuated body weight gain, liver injury, steatosis and reduced level of neutrophil infiltration compared to their HFDE fed WT controls. Importantly, we showed that *Camp^–/–^* mice had significantly increased adipose tissue FGF21/adiponectin production, and increased adipose tissue lipolysis. 

There were no differences between WT and *Camp^–/–^* mice on HFD alone induced liver injury and lipid accumulation in our study. Previously, Tran et al. reported that *Camp^–/–^* mice developed more severe liver steatosis than WT mice after HFD treatment [12]. The reason for this discrepancy is unclear, but we speculate that diet differences may play a role. In our study, we used a HFD that contains cholesterol, whereas the diet Tran et al. used in their study did not. It has been demonstrated that the combination of 15% fat and 1% cholesterol results in severe hepatic steatosis and inflammation [63], and thus the genetic effects of *Camp* deletion could possibly be overwritten in our feeding model. Additionally, the HFD we used contains higher level of sucrose and saturated fatty acids, which could possibly contribute to this discrepancy as well. 

HFD-induced adipose expansion, followed by hypoxia and subsequent adipocyte death, triggers chronic, low-grade inflammation and changes in adipokine expression including adiponectin [64]. We found a dramatic increase in adipose mass and adipocyte size induced by HFDE in WT mice, which were attenuated in *Camp^–/–^* mice. FGF21 regulates the basal secretion of adiponectin and the induction of adiponectin secretion in response to an HFD in adipose tissue, which, in turn, reduces hepatic fat accumulation [27,29,65]. On the other hand, adiponectin mediates the metabolic effects of FGF21 on glucose homeostasis and insulin sensitivity in mice [65]. We showed that HFDE fed *Camp^–/–^* mice had increased FGF21 protein levels in the eWAT compared to WT mice. Importantly, HFDE increased eWAT adiponectin mRNA expression in *Camp^–/–^* but not WT mice, and that *Camp^–/– ^*mice had higher serum adiponectin levels. Additionally, adipose-derived FGF21 can act in an autocrine/paracrine manner to increase the expression of UCP1 and other thermogenic genes in fat tissues [66], leading to increased energy expenditure. In addition, we showed that *Camp^–/–^* mice had increased UCP1 protein level in the BAT, indicating an enhanced energy expenditure when *Camp* is depleted, which may possibly be through FGF21 regulation. Leptin is another important hormone that is predominantly produced by adipocyte, which plays a vital role in energy homeostasis and metabolism [67]. Studies have shown that mice fed with a high-fat diet developed obesity and leptin resistance [68]. Importantly, Hochberg et al. demonstrated in a human study that *CAMP* expression was negatively correlated with leptin level in subcutaneous adipose tissue of obese subjects undergoing a bariatric surgery [69]. It will be interesting to investigate how does the deficiency of CRAMP affect leptin expression and whether it can contribute to the protective effects on fatty liver in our animal model. 

Expression and secretion of both adiponectin and FGF21 are induced in adipose tissue by the activation of PPARγ [70]. We showed that HFDE significantly increased PPARγ protein levels in the eWAT of both WT and *Camp^–/–^* mice, but more robustly in *Camp^–/–^* mice. Importantly, *Camp^–/–^* mice had significantly elevated PPARγ protein in the eWAT compared to WT mice under both CDE and HFDE conditions. PPARγ is activated by various fatty acids and their metabolites [71,72], and its activity is regulated by post-translational modification including phosphorylation [73,74,75] and sumoylation [76,77,78]. Increased sumoylation of PPARγ is associated with reduced transcriptional activity of PPARγ. Previous studies showed that FGF21 prevents sumoylation and restores PPAR activity [28]. It is possible that the deletion of *Camp* may increase PPARγ activity through sumoylation inhibition mediated by FGF21 upregulation, in addition to the increased PPARγ expression on the translational level, enhancing adiponectin production. 

Additionally, we showed in the present study that HFDE significantly increased the mRNA expression of fatty acid transporters, CD36 and FATP2, in WT but not *Camp^–/–^* mice, suggesting that the uptake of lipids by the liver is inhibited when CRAMP is knocked out. NEFA derived from adipose tissue lipolysis is one of the major fat sources that could contribute to hepatic steatosis [16,17]. Previous studies by our group have demonstrated that alcohol-induced adipose lipolysis is mediated by FGF21 through systemic catecholamine release in mice [17]. Eight-weeks of HFD feeding has been shown to increase basal, but blunt epinephrine-stimulated, lipolysis in mouse eWAT and subcutaneous adipocytes, which is mediated by impaired adrenergic receptor signaling, suppression of HSL phosphorylation, and upregulation of ATGL content [79]. It is still unknown whether the combination of HFD and alcohol affects adipose lipolysis, and how it is regulated. Here, we showed that the combination of HFD and binge alcohol greatly suppressed the ATGL protein and phosphorylated HSL in the eWAT of WT mice, which were restored by the deletion of *Camp*. These data indicate an increased eWAT lipolysis with CRAMP depletion, which could possibly contribute to the attenuated adipose hypertrophy in *Camp^–/–^* mice. 

This is the first study demonstrating the involvement of CRAMP in FGF21/adiponectin regulation and adipose lipolysis in a mouse model of HFDE-induced liver injury. A key question remains on how CRAMP expression is regulated in the adipose tissue under HFDE feeding conditions. In a mouse model of *S. aureus* infection, researchers showed that cathelicidin protein and mRNA were strongly induced in the subcutaneous adipocytes, which is associated with an expansion of subcutaneous adipose tissue [80]. In the present study, we found that the addition of acute alcohol did not affect hepatic, but did significantly induce eWAT *Camp* mRNA expression in both CD and HFD-fed WT mice. However, there were no differences in *Camp* mRNA between CDE and HFDE treated WT mice (Figure S6). 

The alcohol-metabolism-generated reactive oxygen species is a hallmark in ALD development and progression [81]. We have showed previously that in a mouse model of binge on chronic alcohol feeding, hepatic *Camp* mRNA was significantly increased in WT mice, and this is associated with an increased reactive oxygen species production [13], indicating that CRAMP might play a role in alcohol metabolism-induced oxidative stress. We found in the present study that hepatic protein level of Cytochrome P450 2E1, a critical enzyme involved in alcohol metabolism was upregulated in both WT and *Camp^–/–^* mice, but to a similar extent under a HFDE treatment (data not shown). Further analysis is needed to better elucidate CRAMP involvement in HFDE-induced liver injury model. 

Collectively (Figure 6F), we have shown in our study that the deletion of *Camp* protects mice against HFDE-induced liver steatosis, injury and inflammation through upregulating FGF21/adiponectin signaling and increasing adipose lipolysis in the eWAT. Additionally, the deletion of CRAMP diminished HFDE-induced hepatic neutrophil infiltration, which is mediated by CRAMP-CXCR2 signaling. 

## Supplementary Materials

The following are available online at https://www.mdpi.com/article/10.3390/cells10123333/s1, Figure S1: Fasting blood glucose level and oral glucose tolerance test (OGTT); Figure S2: Hepatic *Camp* expression and flow cytometry analysis of hepatic immune cells; Figure S3: Serum free fatty acid (FFA) levels; Figure S4: *Fgf21* expression in the eWAT; Figure S5: Hepatic FGF21 protein level and serum FGF21 protein level; Figure S6: *Camp* mRNA expression in the eWAT; Table S1: Primer Sequences for Real-Time RT-PCR analysis (Source: mouse).
