# Supplementary material for: Deficiency of Cathelicidin Attenuates High-Fat Diet Plus Alcohol-Induced Liver Injury through FGF21/Adiponectin Regulation
Source: Cells. 2021 Nov 27;10(12):3333. doi: 10.3390/cells10123333 (PMC8699208; doi:10.3390/cells10123333)
Supplement: Supplementary file 1 [file cells-10-03333-s001.zip › cells-1463734-supplementary.pdf]

SUPPLEMENTAL INFORMATION

Li, et al.

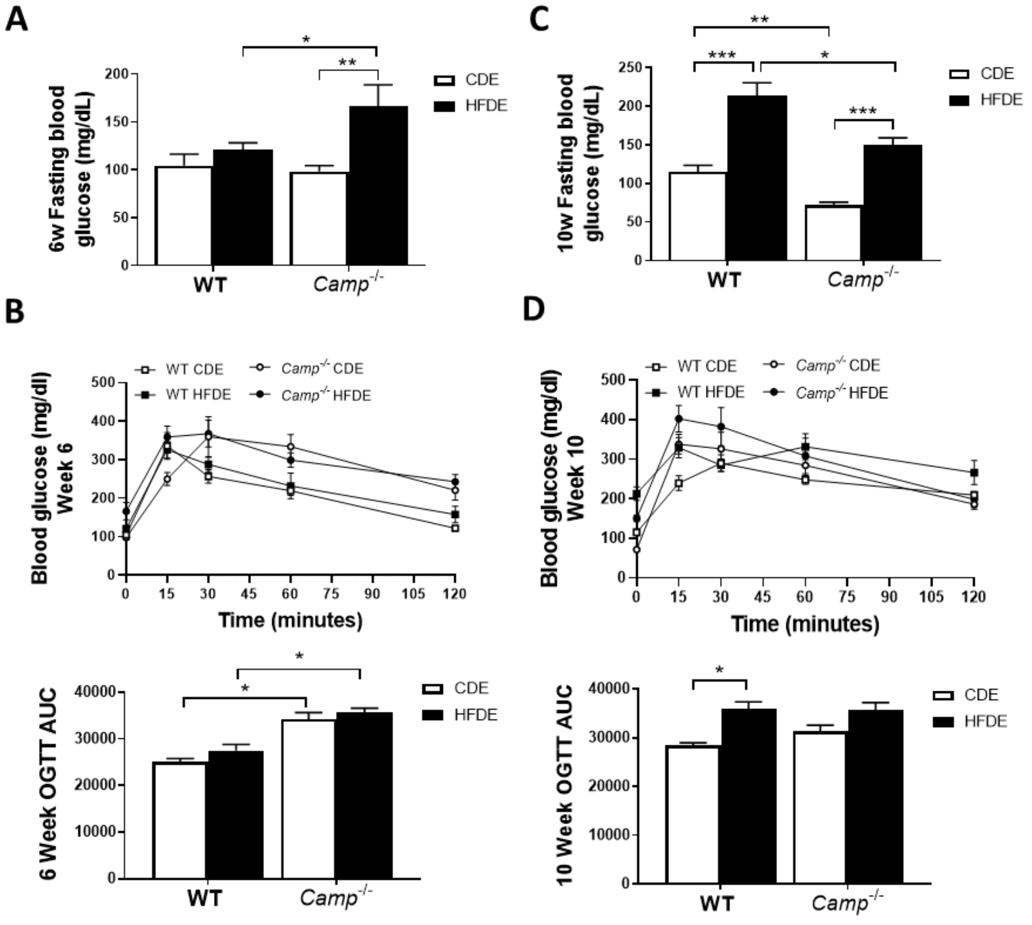

Figure S1. Fasting blood glucose level and oral glucose tolerance test (OGTT). Fasting blood glucose level on week 6 (A) and week 10 (C). OGTT results at week 6 (B) and week 10 (D) shown as blood glucose levels of mice after glucose gavage (upper panel) and the AUC of blood glucose change (lower panel). Data are expressed in Mean ± SEM (n=5 mice/group). $*p \leq 0.05$, $**p \leq 0.01$, $***p \leq 0.001$.

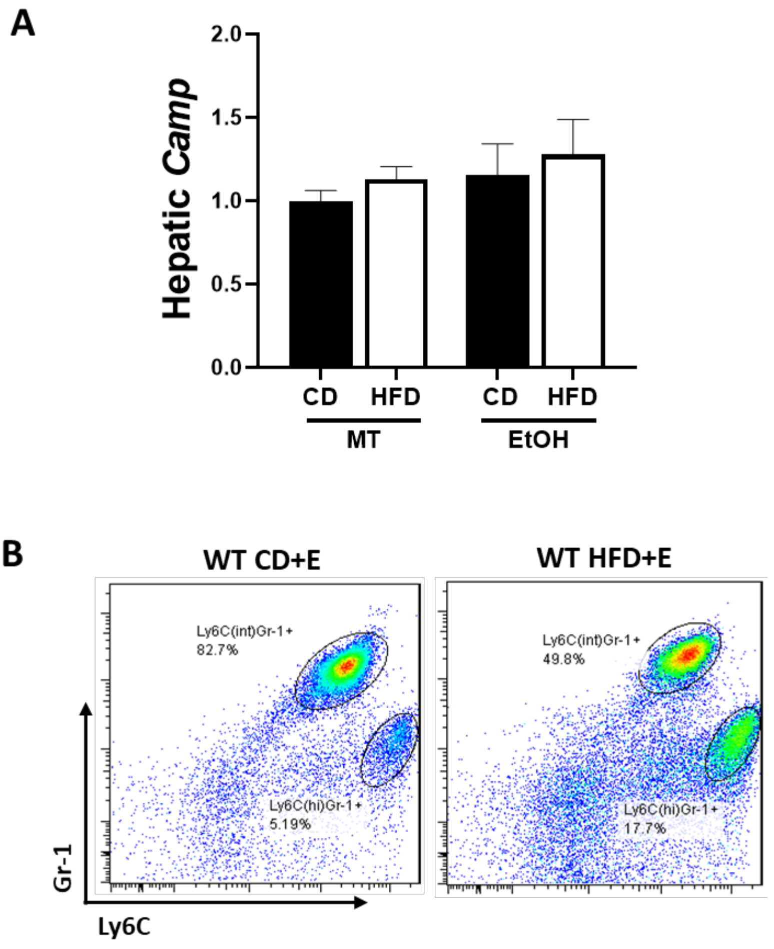

Figure S2. Hepatic *Camp* expression and flow cytometry analysis of hepatic immune cells. (A) *Camp* mRNA expression in the liver. CD: control diet; HFD: high fat diet; Ctrl: control; EtOH: ethanol. (B) Flow cytometry analysis of gated CD11b and Gr1 positive liver leukocytes by antibodies Ly6C. CD11b+Gr1+ Ly6C(int) neutrophils and CD11b+Gr1+ Ly6C(hi) inflammatory monocytes were shown. Data are expressed in Mean ± SEM (n=3-5 mice/group).

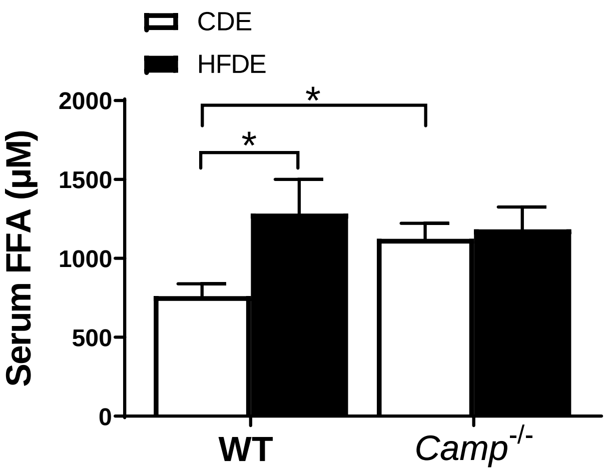

Figure S3. Serum free fatty acid (FFA) levels. Data are expressed in Mean ± SEM (n=7 mice/group). *p≤0.05.

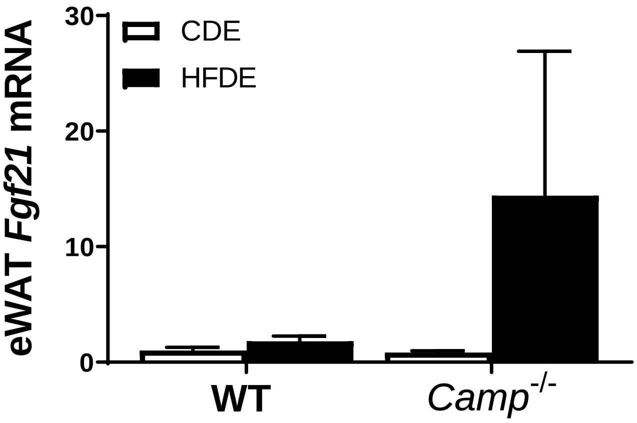

Figure S4. *Fgf21* expression in the eWAT. Data are expressed in Mean ± SEM (n=5 mice/group).

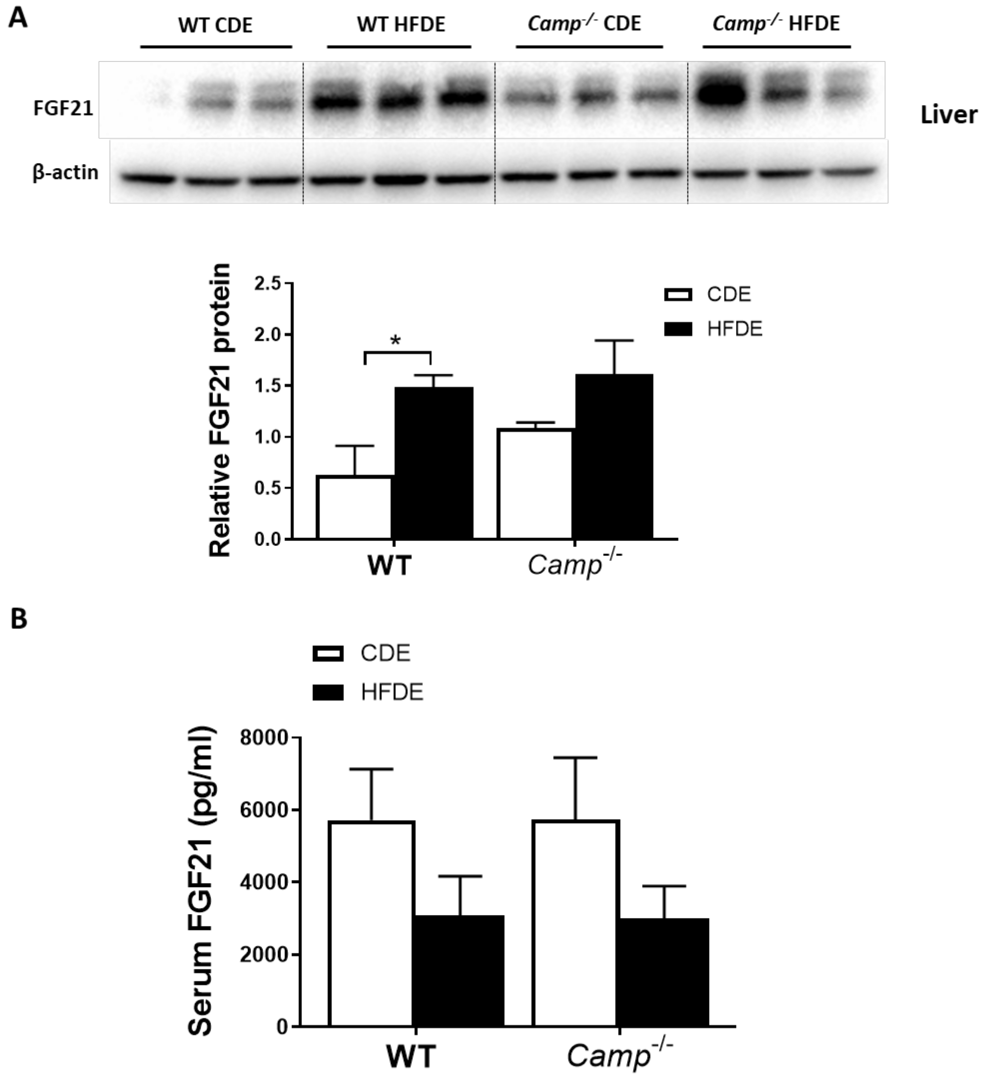

Figure S5. **Hepatic FGF21 protein level and serum FGF21 protein level.** (A) Hepatic FGF21 protein levels (upper panel) and its quantification (lower left panel), representative immunoblots were shown. (n=5 mice/group). (B) Serum FGF21 concentrations, n=6-10. Data are expressed in Mean ± SEM. *$p \leq 0.05$.

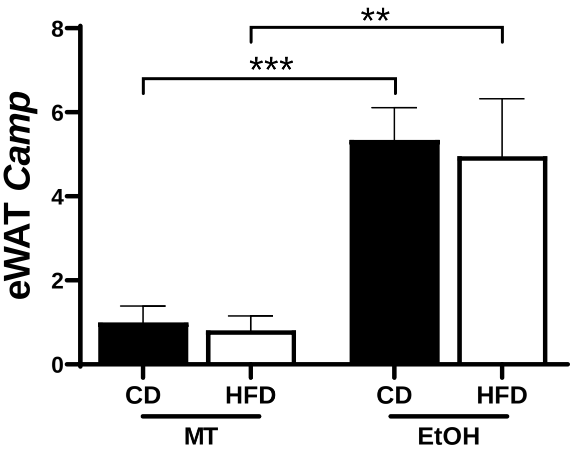

Figure S6. *Camp* mRNA expression in the eWAT. CD: control diet; HFD: high fat diet; Ctrl: control; EtOH: ethanol. Data are expressed in Mean ± SEM (n=5-7 mice/group). **p<0.01, ***p<0.001.

Table S1. Primer Sequences for Real-Time RT-PCR analysis (Source: mouse)

Gene	Sequences (Forward/Reverse 5'-3')		NCBI Accession Number
Srebp1c	CGGAAGCTGTCGGGGTAG	GTTGTTGATGAGCTGGAGCA	NM_001358315.1
Fasn	ATGCACAGAAGGGAAGGAGT	CAGGGTGCAGTTTGTTTCCA	NM_007988.3
Cxcr2	CACCGATGTATACCTGCTGA	ACGCAGTACGACCCTCAAAC	NM_009909.3
Mcp1	ACCACAGTCCATGCCATCAC	TTGAGGTGGTTGTGGAAAAG	NM_011333.3
Cxcl2	ATCCAGAGCTTGAGTGTGACG	GTTAGCCTTGCCTTTGTTCAG	NM_009140.2
F4/80	TATCTTTTCCTCGCCTGCTTC	CACCACCTTCAGGTTTCTCAC	X93328.1
Cxcl1	ACTGCACCCAAACCGAAGTC	TGGGGACACTTTTAGCATCTT	NM_008176.3
Cd36	ATGGGCTGTGATCGGAACTG	TTTGCCACGTCATCTGGGTTT	NM_001159558.1
Fatp2	AACACATCGCGGAGTACCTG	CTCAGTCATGGGCACAAATG	AF072757.1
Fgf21	CCTCTAGGTTTCTTTGCCAACAG	AAGCTGCAGGCCTCAGGAT	NM_020013.4
Pparg	CGCTGATGCACTGCCTATGA	AGAGGTCCACAGAGCTGATTCC	NM_001308352.1
Camp	TCTCTACCGTCTCCTGGACCTG	CCACATACAGTCTCCTTCACTCG	NM_009921.2
18S rRNA	GTAACCCGTTGAACCCCATT	CCATCCAATCGGTAGTAGCG	M35283.1
